# Síndrome Respiratória Aguda Grave (SRAG) Causada por COVID-19: Um Fator Regional

**DOI:** 10.36660/abc.20210803

**Published:** 2021-11-01

**Affiliations:** 1 Universidade Federal de Alagoas Arapiraca AL Brasil Universidade Federal de Alagoas, Arapiraca, AL - Brasil; 2 Centro Universitário Tiradentes Maceió AL Brasil Centro Universitário Tiradentes, Maceió, AL - Brasil

**Keywords:** Doenças Cardiovasculares, COVID-19, Síndrome Respiratória Aguda Grave

A doença causada pelo coronavírus SARS-CoV-2, identificada na China no final de 2019 e denominada COVID-19 (do inglês *coronavirus disease*), foi declarada como uma pandemia pela Organização Mundial da Saúde (OMS) em março de 2020, acumulando mais de 20 milhões de casos confirmados no Brasil, e mais de 583 mil mortes causadas por complicações da doença, até o final da semana epidemiológica 35, em 04 de setembro de 2021.^1–33^ Segundo a OMS, o Brasil é o terceiro país com mais casos confirmados da SARS-CoV-2, ficando atrás somente dos Estados Unidos da América e Índia,^1–3^ a qual é também considerada um país emergente e com problemas socioeconômicos semelhantes aos enfrentados pela população brasileira.

Os sintomas da infecção pelo SARS-CoV-2 podem variar desde uma Síndrome Gripal (SG), com sinais e sintomas leves como febre, tosse, congestão nasal e fadiga, até uma Síndrome Respiratória Aguda Grave (SRAG), com sintomas como dispneia, saturação de O2 ≤ 93%, frequência respiratória ≥ 30 respirações por minuto, pressão arterial de oxigênio (PaO2) /fração inspirada de oxigênio (FIO2) < 300, linfopenia e edema alveolar.^4,5^ No Brasil, foram notificados cerca de 1 775 816 casos de SRAG por COVID-19, sendo que 32,2% dos casos evoluíram para óbito.^6^ Entre os 342.636 óbitos de SRAG por COVID-19 notificados em 2021 até a semana epidemiológica 36, 59,5% apresentavam pelo menos uma comorbidade, sendo as doenças cardíacas, doenças cerebrovasculares, hipertensão arterial sistêmica e diabetes *mellitus* (DM), as mais frequentes.^6,7^

Diante da alta prevalência das doenças cardiovasculares (DCVs), principalmente entre os idosos e sua associação com o desenvolvimento de quadros graves da doença, Paiva et al.,^8^ tiveram como objetivo descrever a prevalência de SRAG por COVID-19 e analisar os fatores associados a essa condição em adultos e idosos com DCV crônica no Brasil até a 30^a^ semana epidemiológica de 2020. Os autores utilizaram dados fornecidos pelo Sistema de Informação de Vigilância Epidemiológica da Gripe (SIVEP-Gripe) e incluíram pessoas acima de 18 anos com DCVs hospitalizados com SRAG. Neste estudo, foram incluídos 116 343 pacientes, sendo que 61,9% foram diagnosticados com SRAG por COVID-19. Os autores encontraram características clínicas, sinais e sintomas semelhantes aos descritos na literatura.^9^ Os fatores associados a SRAG por COVID-19 foram febre, tosse, internação em unidade de terapia intensiva, uso de suporte ventilatório invasivo e não invasivo, e infecção nosocomial. Os resultados do estudo apontam maior prevalência de SRAG na faixa etária acima de 60 anos e na região Sudeste do Brasil.

Os resultados de Paiva et al.^8^ refletem o que já foi apontado em estudos anteriores.^10^ Destaca-se como principal contribuição, os dados de prevalência de SRAG por COVID-19 analisada por cada variável. Observou-se que a maior parte dos casos de SRAG no Nordeste e no Norte foram ocasionados pela COVID-19, um padrão diferente do Sul do país, onde somente 39,9% dos casos foram ocasionados pela COVID-19. Esses resultados demonstram que a prevalência de SRAG por COVID-19 tem um fator regional, refletindo a diferença de acesso ao sistema de saúde e a desigualdade social entre as regiões do Brasil. Um estudo publicado por Baggio et al.,^11^ em Alagoas, mostrou alta taxa de incidência de COVID-19 nos municípios com os melhores índices de desenvolvimento humano, assim como, nos municípios com alta vulnerabilidade social. Entretanto, as maiores taxas de mortalidade foram observadas nos municípios mais pobres. No norte do país, foram observadas as maiores prevalências de comorbidades em pessoas internadas pela COVID-19, o que explica parcialmente o maior risco de morte nessa região.^12,13^ Somado a isso, precisamos levar em consideração o perfil de morbidade, a infraestrutura de saúde (número de leitos, profissionais capacitados), o acesso aos testes de diagnóstico e o tratamento em UTI com processos qualificados e seguros ao paciente.

A fim de aprimorar a análise realizada pelos autores seria interessante a inclusão dos dados sobre a presença de outras comorbidades, além das DCVs, como nível de atividade física prévia e acompanhamento médico para as DCVs. Além disso, a inclusão de outras comorbidades e o nível de atividade física na análise multivariada, permitiria estabelecer a curva de sobrevivência e mortalidade associado às outras comorbidades, como DM, obesidade, sedentarismo entre outros, as quais são comuns nessa população e possuem um papel importante no desenvolvimento de quadros graves da COVID-19.
