# Supplementary material for: Síndrome Respiratória Aguda Grave (SRAG) Causada por COVID-19: Um Fator Regional
Source: Arq Bras Cardiol. 2021 Nov 1;117(5):976–7. [Article in Portuguese] doi: 10.36660/abc.20210803 (PMC8682097; doi:10.36660/abc.20210803)
Supplement: Translation

# Translation: Severe Acute Respiratory Distress Syndrome (ARDS) Caused by COVID-19: A Regional Factor

**Affiliations:** 1 Arapiraca AL Brazil Universidade Federal de Alagoas, Arapiraca, AL - Brazil; 2 Maceió AL Brazil Centro Universitário Tiradentes, Maceió, AL - Brazil

**Keywords:** Cardiovascular Diseases, COVID-19, Severe Acute Respiratory Syndrome

The COVID-19 (Coronavirus disease-19), a disease caused by the SARS-CoV-2, was identified in China at the end of 2019 and announced as a pandemic by the World Health Organization (WHO) in March 2020, with more than 20 million cases confirmed in Brazil, and more than 583 thousand deaths from complications of the disease, by the end of epidemiological week 35, on September 4, 2021.^1–3^ According to the WHO, Brazil is the third country in number of cases, with the USA on the top of the list, followed by India,^1–3^ which is also considered an emerging country with problems similar to those faced by the Brazilian population.

Symptoms of the SARS-CoV-2 infection may vary from a flu-like syndrome, with mild symptoms and signs like fever, cough, nasal congestion and fatigue, to acute respiratory distress syndrome (ARDS), with symptoms including dyspnea, O_2_ saturation ≤ 93%, respiratory rate ≥ 30 breaths per minute, partial pressure of oxygen (PaO2)/fraction of inspired oxygen (FiO2)< 300, lymphopenia, and alveolar edema.^4,5^ Nearly 1,775,816 cases of ARDS secondary to COVID-19 were recorded in Brazil and 32.2% of the cases progressed to death.^6^ Of the 342,636 deaths for ARDS due to COVID-19 recorded up to the epidemiological week 36 in 2021, 59.5% had at least one comorbidity, with heart diseases, cerebrovascular diseases, systemic arterial hypertension and diabetes mellitus (DM) as the most common ones.^6,7^

Considering the high prevalence of cardiovascular diseases (CVDs), especially in the elderly, and its association with more severe cases of the disease, Paiva et al.^8^ aimed at describing the prevalence of ARDS due to COVID-19 and evaluating the factors associated with this condition in adults and elderly with chronic CVD in Brazil up to the 30th epidemiological week in 2020. The authors used data from the epidemiological surveillance system on influenza (*Sistema de Informação de Vigilância Epidemiológica da Gripe*, SIVEP-Gripe) and included individuals with CVDs, aged over 18 years old, hospitalized for ARDS. In their study, 116,343 patients were included, 61.9% of them received a diagnosis of ARDS due to COVID-19. The authors found clinical features, signs and symptoms that were similar to those described in the literature.^9^ Factors associated with ARDS due to COVID-19 were fever, cough, intensive care unit admission, use of invasive and non-invasive ventilation, and nosocomial infection. The results indicated a higher prevalence of ARDS in individuals aged over 60 years from the southeast of Brazil.

The results of Paiva et al.^8^ reflect what has already been pointed out in previous studies.^10^ The main contribution of the study is the data on the prevalence of ARDS by COVID-19 analyzed by each variable. The authors found that most cases of ARDS in the north and northeast regions of Brazil were caused by COVID-19, which differed from the cases in the south of the country, where 39.9% of the cases were caused by COVID-19. These data indicate that the prevalence of COVID-19-related ARDS has a regional factor, and reflect the difference in the access to healthcare and social inequalities among the regions. A study published by Baggio et al.,^11^ in Alagoas, showed a high incidence of COVID-19 in the cities with the highest human development indexes, as well as in those with high social vulnerability. However, the highest mortality rates were found in the poorest cities. The highest prevalence of comorbidities was observed in patients hospitalized for COVID-19, which explain in part the highest risk of death in this region.^12,13^ Besides, one should consider the morbidity profile, healthcare infrastructure (number of beds, trained professionals), and access to diagnostic tests and intensive therapy with qualified and safe processes by the patients.

The inclusion of data about the presence of other comorbidities, in addition to CVDs, like physical activity level and clinical follow-up for CVDs, would aid to improve the analysis conducted by the authors. Also, the inclusion of other comorbidities and physical activity level to the multivariate analysis would allow the establishment of survival and mortality curves associated to other comorbidities, including DM, obesity, sedentary lifestyle, among other common conditions that play an important role in the development of severe cases of COVID-19.
